# Transcriptomic Analysis of Carboxylic Acid Challenge in *Escherichia coli*: Beyond Membrane Damage

**DOI:** 10.1371/journal.pone.0089580

**Published:** 2014-02-28

**Authors:** Liam A. Royce, Erin Boggess, Yao Fu, Ping Liu, Jacqueline V. Shanks, Julie Dickerson, Laura R. Jarboe

**Affiliations:** 1 Department of Chemical and Biological Engineering, Iowa State University, Ames, Iowa, United States of America; 2 Department of Electrical and Computer Engineering, Iowa State University, Ames, Iowa, United States of America; 3 Interdepartmental Microbiology Program, Iowa State University, Ames, Iowa, United States of America; Arizona State University, United States of America

## Abstract

Carboxylic acids are an attractive biorenewable chemical. Enormous progress has been made in engineering microbes for production of these compounds though titers remain lower than desired. Here we used transcriptome analysis of *Escherichia coli* during exogenous challenge with octanoic acid (C8) at pH 7.0 to probe mechanisms of toxicity. This analysis highlights the intracellular acidification and membrane damage caused by C8 challenge. Network component analysis identified transcription factors with altered activity including GadE, the activator of the glutamate-dependent acid resistance system (AR2) and Lrp, the amino acid biosynthesis regulator. The intracellular acidification was quantified during exogenous challenge, but was not observed in a carboxylic acid producing strain, though this may be due to lower titers than those used in our exogenous challenge studies. We developed a framework for predicting the proton motive force during adaptation to strong inorganic acids and carboxylic acids. This model predicts that inorganic acid challenge is mitigated by cation accumulation, but that carboxylic acid challenge inverts the proton motive force and requires anion accumulation. Utilization of native acid resistance systems was not useful in terms of supporting growth or alleviating intracellular acidification. AR2 was found to be non-functional, possibly due to membrane damage. We proposed that interaction of Lrp and C8 resulted in repression of amino acid biosynthesis. However, this hypothesis was not supported by perturbation of *lrp* expression or amino acid supplementation. *E. coli* strains were also engineered for altered cyclopropane fatty acid content in the membrane, which had a dramatic effect on membrane properties, though C8 tolerance was not increased. We conclude that achieving higher production titers requires circumventing the membrane damage. As higher titers are achieved, acidification may become problematic.

## Introduction

There has been a substantial interest in using microbial fatty acid biosynthesis as a platform for a variety of biorenewable chemicals [1]–[4]. However, there are challenges associated with harnessing the fatty acid biosynthesis pathway for producing chemicals at industrially relevant titers, productivities, and yields. For example, it has been noted by multiple researchers that product toxicity is a major problem for the microbial production of carboxylic acids [5]–[9].

Microbial tolerance of inhibitors, either present in the plant-derived feedstock or a toxic desired product, is a common problem in the fermentative production of biorenewable fuels and chemicals [10]–[12]. Knowing the mechanism of inhibition can enable rational design strategies for addressing tolerance. Transcriptome analysis is one method for identifying these mechanisms [7], [10], [13], [14]. It is relatively well-established that one of the major effects of short chain carboxylic acid toxicity is membrane damage, largely due to the hydrophobic nature of the carbon chain [15]. It is also well-accepted that exogenous challenge with carboxylic acids can cause intracellular acidification, interfering with cellular function and imposing an ATP burden [16]–[19]. Our previous work quantified the effect of octanoic acid (C8) on membrane integrity, fluidity, hydrophobicity and composition [5] and we concluded that diffusion of octanoic acid into the membrane impairs its function. Here we used transcriptomic analysis of exogenous octanoic acid challenge to identify and quantify other mechanisms of inhibition, as well as exploring strategies for improving tolerance.

## Materials and Methods

### Strains and growth conditions

*Escherichia coli* strain K-12 MG1655 was obtained from ATCC (Manassas, VA, USA) (Table 1). All strains were grown in 25 mL MOPS minimal media [20] with 2% dextrose in 250 mL baffled flasks at 37°C. Overnight cultures were diluted to an optical density of 0.05 at 550 nm (OD_550_) for specific growth measurements and RNA extraction, and diluted to 0.1 for intracellular pH, γ-amino butyric acid, and membrane lipid composition measurements. 4 M C8 stock solutions were prepared in 100% ethanol. The concentration of ethanol used in these experiments did not have a significant impact on growth (*data not shown*). Media with octanoic acid were pH-adjusted to 7.0 with 2 M potassium hydroxide. The specific growth rate was determined by measuring the OD_550_ during the exponential phase, as previously described [5].

**Table 1:** Strains and plasmids used in this study.

Strain and plasmids	Genotype	Reference
MG1655 ATCC#700926	*F- lambda- ilvG- rfb-50 rph-1*	Wildtype
ML103	MG1655Δ*fadD*	[21]
Δ*cfa*	MG1655Δ*cfa*	This reference
*cfa*++	MG1655+pCA-*cfa*	[23]
Δ*lrp*	MG1655Δ*lrp*	This reference
*lrp*++	MG1655+pCA-*lrp*	[23]
pJDT1	pBad24-TorA-GFPmut3*	[22]
pXZ18Z	pTrc99a-acyl Thioesterase *R. communis-fabZ*	[25]
pZS-GFP	pZS-TorA-GFPmut3*	[24] and this reference

The carboxylic acid production strain ML103 [21] was obtained from Ka-Yiu San (Rice University). This strain was grown in 25 mL M9 with 1.5% dextrose in 250 mL baffled flasks at 30°C with the appropriate antibiotics and inducers, as described below.

### Plasmids

pJDT1 [22],obtained from Anja Nenninger (University of London), was induced by 200 µM L-arabinose and 100 µg/mL ampicillin was used for maintenance. pCA-*cfa* and pCA-*lrp* [23], obtained from Ramon Gonzalez (Rice University), were induced by 0.5 mM isopropyl β-D-1-thiogalactopyranoside (IPTG) and 25 µg/mL chloramphenicol was used for maintenence. pZS-GFP was cloned using the Gibson Assembly Cloning kit (New England Biolabs, Ipswich, MA, USA), with pZS [24] as the backbone and the GFP gene from pJDT1. pZS-GFP was induced by using 50 ng/mL anhydrotetracycline (Fisher Scientific, Hampton, NH, USA) and 25 µg/mL chloramphenicol was used for maintenance. pXZ18Z [25], obtained from Ka-Yiu San (Rice University), was induced by 100 µM IPTG and 100 µg/mL ampicillin was used for maintenance (Table 1).

### Transcriptomics sample preparation

*E. coli* MG1655 was grown to midlog (OD_550_∼0.8) with or without 10 mM octanoic acid and cells were harvested for RNA purification. Briefly, cells were harvested by swirling in a dry ice/ethanol water bath until cold and then centrifuged at 5,000 g, 20 min. at 4°C; the resulting cell pellets were stored in RNA Later solution (Life Technologies, Carlsbad, CA) at −80°C. RNase AWAY solution (Life Technologies) was used to remove contaminating RNase. The RNeasy Mini Kit (Qiagen, Venlo, Netherlands) was used to isolate total RNA, which was then incubated at 37°C with RNaseOut (New England Biolabs) and DNaseI (New England Biolabs) according to the manufacture's protocol for 1 hour. The samples were mixed with saturated phenol/chloroform (pH = 4.5) (Life Technologies) and precipitated with ethanol and 30 µL 3 M sodium acetate (pH = 5.5) (Fisher Scientific) overnight at −80°C. After precipitation, the tubes were centrifuged for 30 min. at 16,873 g, 4°C. The RNA pellet was washed twice with 70% ethanol and dried under vacuum for 30 min. RNA quality was confirmed on a 1% TAE gel. The RNA was hybridized to an Affymetrix GeneChip *E. coli* Genome 2.0 Array, from which the sample was prepared with the Affymetrix protocols (Affymetrix, Santa Clara, CA) and analyzed on a ProScanArray HT Microarray Scanner (Perkin Elmer, Waltham, MA) at the DNA facility of Iowa State University Office of Biotechnology. The Affymetrix GeneChip *E. coli* Genome 2.0 Array contains 10,208 probes for genes in four strains of *E. coli*. The data is available in the Gene Expression Omnibus database, accession number: GSE53140.

### Transcriptomic data normalization and analysis

Background adjustment, normalization, and summarization calculations were performed in MATLAB v. R2012b (MathWorks, Natick, MA, USA) using GCRMA [26]. Using pooled data, permutation t-tests (10,000 permutations) were performed to identify transcripts with statistically significant changes in abundance (q-value <0.05). Additionally, a fold change (log_2_(treatment/control)) cutoff of ±1 was applied to identify genes that demonstrated a sufficient magnitude of variation between experimental conditions. Probes not annotated as MG1655, but orthologous to *E. coli* strain MG1655 were included. Probes relating to other species were excluded from analysis. Additionally, overrepresentation of Gene Ontology terms associated with significant expression perturbations was examined and visualized using the Cytoscape tool [27] and BiNGO [28].

### Transcription factor analysis

The transcription factor analysis was performed using NCA algorithm, and based on reconstructed regulatory networks using RegulonDB [29] and the predicted TFA information as previously described [30].

### Intracellular pH measurements

*E. coli* MG1655 carrying the pBad24-TorA-GFPmut3* (pJDT1) plasmid [22] was grown in 100 mL potassium-modified Luria broth (LBK) [31] media in a 250 mL baffled flask from OD_550_ 0.05 to midlog (OD_550_∼0.8) with 100 µg/mL ampicillin and 200 µM L-arabinose at 37°C in an orbital incubator shaker at 150 rpm. The cells were harvested at 5,000 g, 4°C for 20 min. and resuspended in phosphate buffered saline (pH = 7.0) diluted to the working concentration (PBS, 10X powder concentrate, Thermo Fisher Scientific, Waltham, MA) without a carbon source to an OD_500_ = 0.4. A calibration curve was generated using sodium benzoate, according to [32]. *E. coli* cell suspensions were challenged with C8 or HCl and pH-adjusted to 7.0 before measurement. The intracellular pH was measured by fluorescence intensity using a Synergy 2 Multi-Mode microplate reader from BioTek with sterile black-bottom Nunclon delta surface 96-well plates in the W.M. Keck Metabolomics Research Laboratory at Iowa State University. The plates were analyzed at 30°C in 100 µL aliquots, using the following filters: excitation filter = 485/20 nm, emission filter = 528/20 nm.

For the amino acid supplementation studies, MG1655+pJDT1 was grown overnight in MOPS media as described above. The cells were harvested at 5,000 g, 4°C for 20 min. and resuspended in MOPS with 0.2 mM L-arabinose and incubated at 37°C for 3 h with or without amino acids (arginine and/or glutamate) and C8 at equimolar concentrations of 10 mM. The cells were harvested as treated as above to measure the intracellular pH.

For the carboxylic acid production study, *E. coli* ML103+pXZ18Z+pZS-GFP was grown as described above. The intracellular pH was measured as described above, except the cells were not challenged with exogenous carboxylic acids prior to measurement.

### γ-amino butyric acid measurements

MG1655 was grown to midlog (OD_550_∼0.8) as described above with 10 mM C8 and/or 10 mM glutamate added to the growth media. Measurement of γ-amino butyric acid was performed according established protocols [33], [34]. All chemicals for this assay were purchased from Sigma-Aldrich, St. Louis, MO. Clear UV-transparent microplates were purchased from Corning (Corning, NY).

### Membrane lipids analysis

The protocol was performed according to [5]. Briefly, cells were grown to midlog (OD_550_∼0.8) as described above, harvested at 5,000 g, 4°C for 20 min, and then challenged with different concentrations of C8 for 3 h in MOPS pH 7.0 with 2% dextrose. After challenge, the cells were harvested for membrane lipid analysis.

### Proton motive force simulations

The total electrochemical force was used to calculate the proton motive force (PMF):
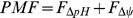
(1)and the Nernst potential was used to calculate the contributing forces:
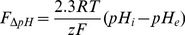
(2)

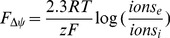
(3)where 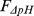 is the electrochemical force-associated pH-gradient, 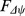 is the electrochemical force-associated ion-gradient, R is the gas constant, T is the temperature, z is the molar ion charge, and F is Faraday's constant. Intracellular and extracellular ion abundances are represented by ions_i_ and ions_e_, respectively. Use of these equations have been previously reviewed [35], [36].

### Statistical analysis

The p-values were obtained using one-way ANOVA and Tukey-Kramer pairs analysis with the JMP v/8.02 statistical program (SAS Institute, Cary, NC, USA). We used a p-value cutoff of 0.05 to determine significance. In the amino acid supplementation survey, we applied the Bonferroni correction factor and adjusted the P-value cutoff to 0.0025.

## Results

### Transcriptome analysis highlights the activation of acid resistance systems

The information gained by transcriptome analysis is valuable in many applications, especially in the area of tolerance for biorenewable chemicals production. Tolerance is a complex phenotype that covers a diverse space in the bacterial genome [37]. For this reason, transcriptomics is a critical tool that allows for a global measurement of the cellular response network of stressors, such as challenge with carboxylic acids. Therefore, we performed transcriptome analysis of *E. coli* MG1655 during mid-log growth in MOPS minimal media with and without 10 mM octanoic acid, where octanoic acid serves as a representative carboxylic acid. Both cultures had an initial pH of 7.0; 10 mM C8 is sufficient to inhibit growth in this condition by 23% [5].

Table 2 lists all significantly perturbed genes (q<0.05) with at least a 2-fold change in response to C8 challenge. The data is also presented graphically in Figure S1 with log_2_ fold changes. Similarly, Table 3 lists all transcription factors with significantly perturbed (p<0.05) regulatory activity, as determined by network component analysis (NCA).

**Table 2:** Differentially expressed genes under C8 stress highlight the acid resistance fitness island.

b1493	*gadB*	0.000	0.015	25.3	AR2 decarboxylase
b3509	*hdeB*	0.000	0.004	21.1	AR1 chaperone protein
b3238	*yhcN*	0.002	0.064	15.2	Acid response
b3510	*hdeA*	0.000	0.000	13.7	AR1 chaperone protein
b4439	*micF*	0.003	0.078	11.9	Antisense RNA regulator of OmpF porin
b3512	*gadE*	0.004	0.115	10.1	TA of AR2
b1492	*gadC*	0.000	0.013	9.3	AR2 antiporter
b3511	*hdeD*	0.001	0.053	8.7	AR1
b1480	*sra*	0.001	0.009	5.1	SP component of the 30S ribosomal subunit
b3508	*yhiD*	0.001	0.017	4.5	density-dependent acid resistance
b0812	*dps*	0.004	0.101	4.4	DNA-binding, iron-collecting, oxidative damage protection/repair
b3517	*gadA*	0.003	0.108	4.4	AR2 decarboxylase
b0485	*ybaS*	0.007	0.144	4.1	Glutaminase- acid shock inducible
b1531	*marA*	0.002	0.067	3.9	Multiple antibiotic resistance TR- organic solvents, oxidative stress
b2013	*yeeE*	0.009	0.157	3.8	Resistance to the DNA-damage
b0287	*yagU*	0.003	0.012	3.5	IMP- acid resistance
b1165	*ymgA*	0.002	0.003	3.5	Biofilm formation- rapid acid treatment
b3516	*gadX*	0.001	0.014	3.5	AR2 TR
b1112	*bhsA*	0.002	0.003	3.3	Influencing biofilm through hydrophobicity and SR
b2012	*yeeD*	0.010	0.106	3.0	Uncharacterized
b1164	*ycgZ*	0.006	0.107	2.9	Cold shock stimulon
b4554 (c4419)	*yibT*	0.001	0.056	2.8	Uncharacterized
b0814	*ompX*	0.004	0.017	2.8	Acid-induced OMP
b4376	*osmY*	0.001	0.023	2.8	Hyperosmotic stress
b3405	*ompR*	0.013	0.163	2.7	OMP TR
b3515	*gadW*	0.026	0.219	2.6	AR2 TR
b2924	*mscS*	0.001	0.028	2.4	IMP mechanosensitive (MS) channel; non-specific transporter
b0850	*ybjC*	0.003	0.011	2.4	marA/SoxS induced
b3024	*ygiW*	0.004	0.014	2.4	IMP transporter, SR
b0775 (c0855)	*bioB*	0.018	0.193	2.3	SAM-dependent biotin synthase
b2425	*cysP*	0.020	0.105	2.2	IMP Sulfate transporter
b1661	*cfa*	0.010	0.110	2.1	Cyclopropane fatty acid synthase
b3160	*yhbW*	0.000	0.015	2.0	Monooxygenase
b4077	*gltP*	0.024	0.213	2.0	Glutamate and aspartate transporter
b3458	*livK*	0.005	0.090	−2.0	leucine transporter
b1194	*ycgR*	0.002	0.018	−2.0	Flagellar motility
b1887	*cheW*	0.003	0.019	−2.0	Flagellar motors
b4484	*cpxP*	0.038	0.116	−2.1	TR, SR of cell envelope
b1075	*flgD*	0.009	0.032	−2.2	Flagella
b1941	*fliI*	0.007	0.031	−2.2	Flagellar export
b0455	*ffs*	0.035	0.220	−2.2	RNA, membrane protein assembly
b1942	*fliJ*	0.002	0.015	−2.5	Flagellar export
b1946	*fliN*	0.012	0.118	−2.5	Flagellar motor
b1921	*fliZ*	0.005	0.096	−2.5	TR flagella
b1922	*fliA*	0.000	0.000	−2.6	Flagella; sigma F factor
b1884	*cheR*	0.001	0.008	−2.8	Chemotaxis sensory transduction system
b1072	*flgA*	0.002	0.017	−3.0	Flagella assembly
b1742 (Z2774)	*ves*	0.010	0.157	−3.0	Cold shock stimulon
b1939	*fliG*	0.001	0.007	−3.2	Flagellar motor
b1945	*fliM*	0.000	0.001	−3.2	Flagellar motor
b1881	*cheZ*	0.012	0.173	−3.3	Chemotaxis sensory transduction system
b1078	*flgG*	0.024	0.223	−3.3	Flagellar motor
b4355	*tsr*	0.004	0.112	−3.4	Chemotaxis proteins
b1073	*flgB*	0.002	0.027	−3.4	Flagellar motor
b1937	*fliE*	0.005	0.045	−3.5	Flagella
b1076 (Z1714)	*flgE*	0.007	0.133	−3.6	Flagellar motor
b0557 (Z1878)	*borD*	0.001	0.011	−3.7	Magnesium stimulon
b1938	*fliF*	0.005	0.109	−3.7	Flagella
b1888	*cheA*	0.015	0.190	−3.7	chemotaxis protein
b1947	*fliO*	0.013	0.154	−3.8	Flagellar export
b1925	*fliS*	0.002	0.033	−3.8	Flagellar export
b1882	*cheY*	0.003	0.033	−3.9	Chemotaxis response regulator
b1071	*flgM*	0.001	0.018	−3.9	Flagella
b3525	*yhjH*	0.003	0.064	−3.9	flagellar motility
b1940 (c2357)	*fliH*	0.001	0.008	−3.9	flagella ATPase inhibitor
b1074	*flgC*	0.001	0.013	−4.0	Flagellar motor
b1944	*fliL*	0.000	0.015	−4.1	Flagella
b1943	*fliK*	0.001	0.003	−4.3	Flagellar export
b1886	*tar*	0.005	0.114	−4.3	Aspartate chemoreceptor
b1924	*fliD*	0.013	0.173	−4.4	Flagella
b1889	*motB*	0.004	0.086	−4.5	Flagellar motor
b1070	*flgN*	0.000	0.000	−5.1	Flagellar export
b1923	*fliC*	0.003	0.095	−5.4	Flagella
b1076	*flgE*	0.004	0.106	−5.8	Flagellar motor
b1890	*motA*	0.000	0.000	−6.0	Flagellar motor
b1566	*flxA*	0.000	0.000	−6.3	Prophage
b1083	*flgL*	0.002	0.083	−9.3	Flagella
b0929	*ompF*	0.000	0.022	−16.0	Transport of sugars, ions, and amino acids <600 daltons
b0553	*nmpC*	0.002	0.069	−16.3	OMP porin associated with the peptidoglycan

Expression ratio for genes significantly perturbed (q<0.05) at least 2-fold are reported as increased abundance (+) or decreased abundance (−). Probes not annotated as MG1655, but homologous to other *E. coli* strains, are annotated as either CFT073 or EDL933, with the corresponding c-number or Z-number, respectively. The table is sorted by the magnitude of the expression ratio and the function categories are derived from the Ecocyc database [68]. Abbreviations: AR1- acid resistance system 1; AR2- glutamate-dependent acid resistance system 2; OMP- outer membrane protein; IMP- inner membrane protein; TA- transcriptional activator; TR- transcriptional regulator; SP- stationary phase; SR- stress response.

**Table 3:** Transcription factor activities significantly changed.

Transcription Factor	ΔTFA	Sensing signal
FruR	−	Fructose 1-phosphate or Fructose 1,6-phosphate
GadE	+	Low pH
Lrp	+	Leucine
MalT	+	Maltodextrin
PutA	−	Proline and FAD
RcsAB	+	Membrane proteins
SoxS	+	Oxidative stress

TFA with a significant change (p<0.05) using NCA algorithm with the RegulonDB regulatory links [29].

Many of the genes with increased expression in response to C8 challenge are related to acid response, response to and regulation of pH, and biofilm formation. Genes with decreased expression are predominantly attributed to chemotaxis, reduced motility, and flagellum assembly. Several genes associated with membrane function and integrity, such as *bhsA* and *cpxP* were also perturbed, where some had increased expression in response to C8 (e.g., *cfa*) and some had decreased expression (e.g., *ompF*). These trends are also reflected in the over-represented Gene Ontology (GO) terms, as listed in Table S1.

**Figure 1:**
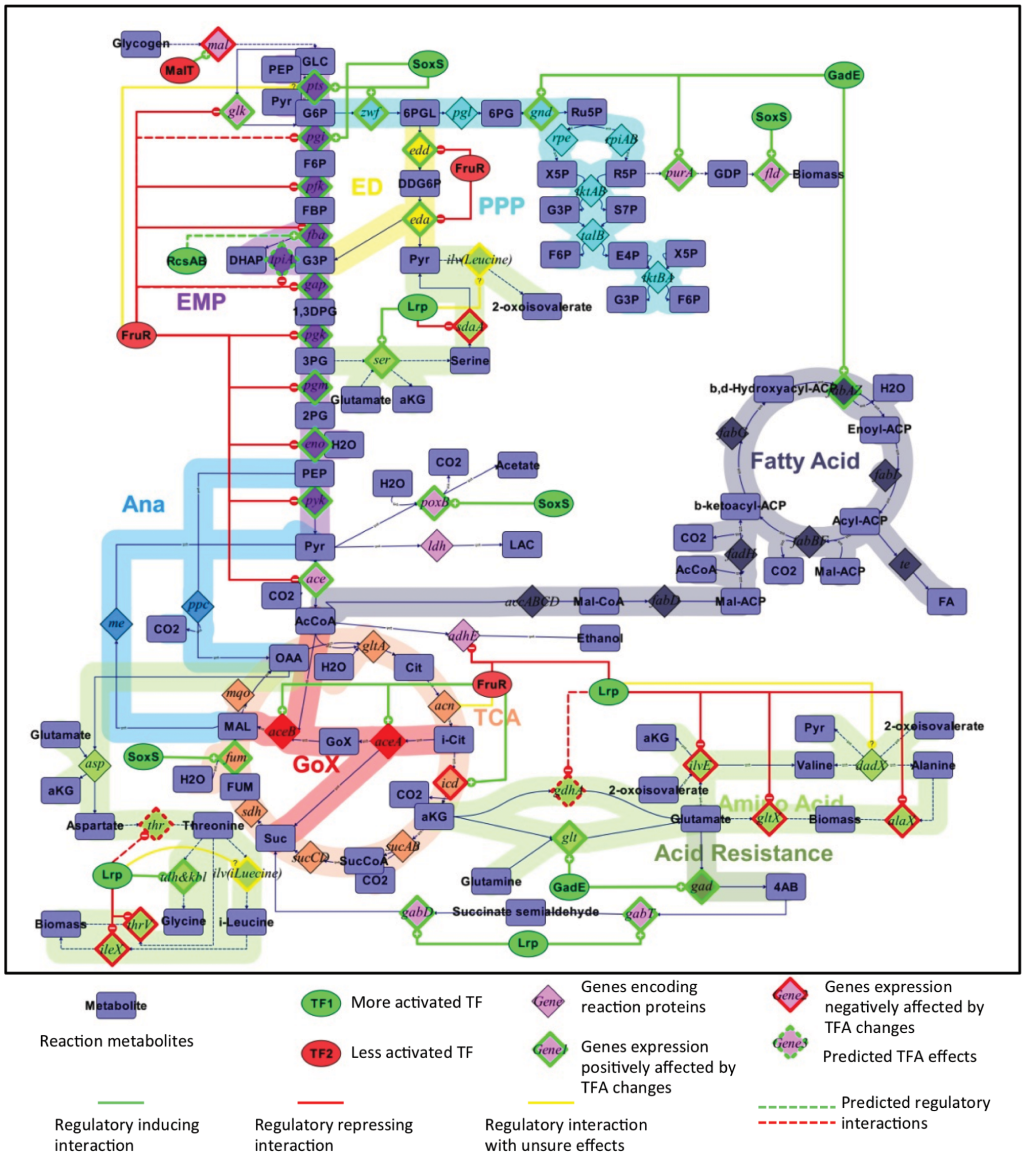
Octanoic acid response network. MG1655 was challenged with sufficient octanoic acid to inhibit growth by 23% (10 mM) in MOPS minimal media at pH 7.0, 37°C, 150 rpm. This diagram shows central metabolism and highlights the regulatory effect of regulators with significantly perturbed activity during C8 challenge, as identified by network component analysis. Dashed lines indicate regulatory connections that were proposed in our previous analysis [30]. Mechanisms for changes in transcription factor activity are discussed in the text.

Similarly, many of the perturbed regulators are also associated with acid response and membrane damage (Figure 1). Most notably, GadE regulates various acid response systems and is primarily controlled at the transcriptional level in response to pH, as previously reviewed [35]. RcsB is known for both association with capsule biosynthesis [38] and acid resistance [39], [40]. It is known that RcsB activity is regulated by membrane-associated partner proteins in response to a variety of extracellular signals [38] and is required for maintaining the appropriate cell shape [41]. RcsB also regulates motility in an H-NS-dependent manner, which could possibly explain the decrease in motility [40]. SoxS activation, while not a direct response to acids or membrane integrity, is most likely due to superoxide production as an effect of membrane damage, as previously reviewed [9].

Activation of Lrp in response to carboxylic acid challenge has been previously described [42]. Specifically, it was reported that the 4-carbon carboxylic acid butyrate binds to Lrp in a manner that mimics leucine. Therefore, it is possible that a similar interaction occurs with octanoic acid, resulting in activation of Lrp during C8 challenge.

The PutA protein shifts between a cytosolic, DNA-binding form and a membrane-associated metabolic enzyme [43], [44]. The switch to the membrane form is dependent upon the availability of proline and FAD; the membrane-associated form then converts proline into glutamate [43]. The abundance of the *putA* transcript is not changed in our dataset. The observed decrease in PutA TFA therefore suggests that the pool of PutA enzymes shifted from the cytosolic form to the membrane-associated form, indicating a possible increase in proline and/or FAD availability. This potential effect is discussed further below. The *putA* gene is also transcriptionally activated by the multiple antibiotic resistance regulator MarA, which is also activated in our transcriptome data [45].

FruR and MalT are both responsive to metabolites in the early steps of glycolysis. FruR DNA-binding activity is decreased by the metabolites fructose-1-phosphate and fructose-1,6-phosphate [46]; decreasing FruR activity suggests increased abundance of one or both of these metabolites. MalT is the regulator specific to catabolism of maltose, maltotriose, and starch containing maltose sugars [47]. Several factors may be involved in the activation of MalT. Intriguingly, MalT activity can be stimulated by increased intracellular maltotriose [48]. This maltotriose can be produced from unphosphorylated intracellular glucose [48]. This unphosphorylated glucose could come from glycogen or possibly enter through the damaged cellular membrane; we have previously quantified this membrane “leakiness” using Mg^2+^ as a reporter molecule [5]. There are other potential sources of unphosphorylated glucose, such as the galactose transporter [48].

Two of the most often cited means of carboxylic acid toxicity are intracellular acidification and membrane damage [18], and many of the genes and regulators perturbed in our dataset can be attributed to these two effects. Membrane damage in this condition has recently been quantified [5]. However, the acidification effect remains relatively undercharacterized.

### Octanoic acid lowers the intracellular pH

**Figure 2:**
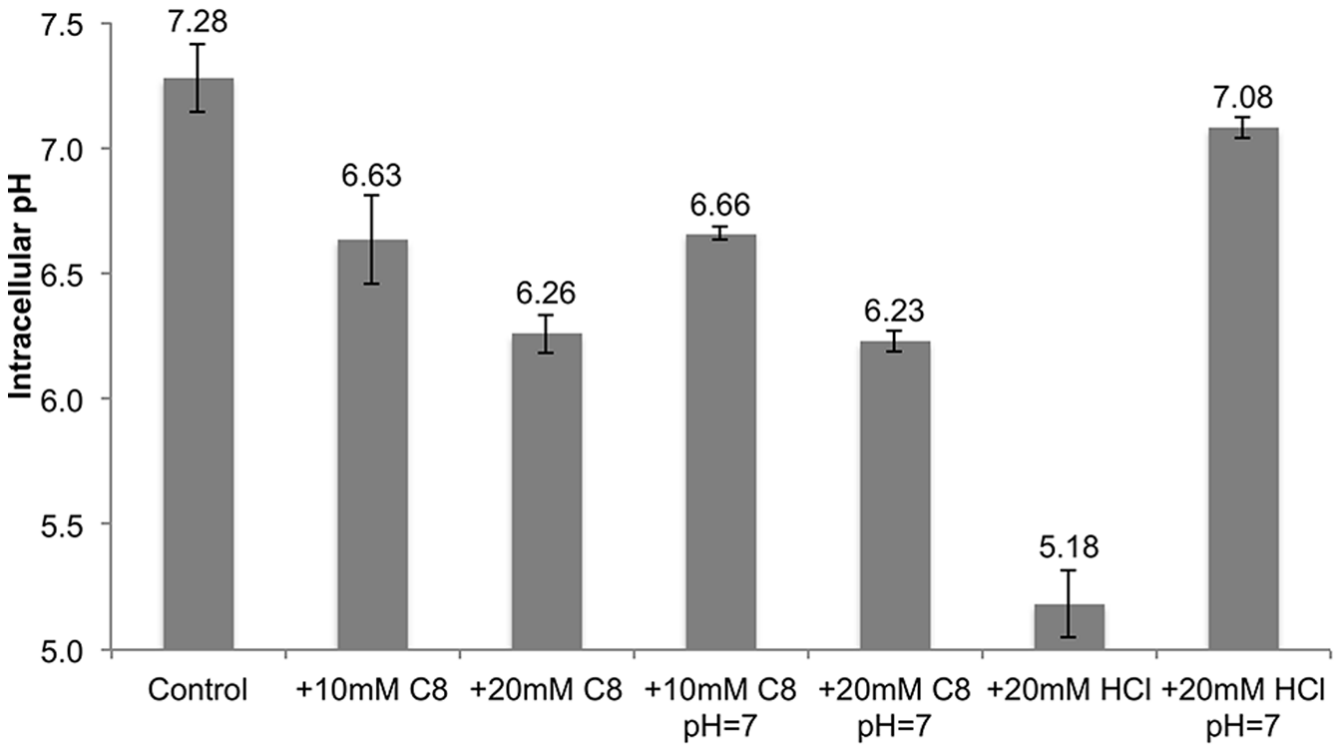
Octanoic acid challenge decreases the intracellular pH. E. coli MG1655 pJTD1 was grown to midlog in minimal media at pH(HCl). Values are the average of 4 biological replicates, with error bars indicating the standard deviation.

We used a pH-dependent modified green fluorescent protein [32] to verify and quantify the acidification effect of carboxylic acid challenge at neutral pH. We used the familiar example of HCl challenge as a positive control for this system. Addition of 20 mM HCl without readjusting media pH leads to a severe decrease in intracellular pH to 5.32 (Figure 2). As expected, adjustment of media pH back to neutral after addition of HCl is associated with the appropriate near-neutral intracellular pH. Contrastingly, addition of 10 or 20 mM C8, even with adjustment of media pH to 7.0, results in a significant (p<0.05) and dose-dependent drop in intracellular pH (Figure 2). This data not only confirms the acidification effect of C8 challenge in neutral media, but also quantifies this effect.

This acidification is consistent with the known permeability of the cell membrane by the neutral protonated form of octanoic acid. Once this neutral form permeates the cell membrane, it is able to deprotonate inside the cell and thereby decrease the intracellular pH [5]. Normally, the intracellular pH is slightly basic in order to maintain proper membrane transport properties in a neutral pH environment. *E. coli* uses this pH gradient as a means of nutrient and waste transport. The pK_a_ of C8 is 4.89 [49]; at pH 7.0; the unprotonated form is dominant in the bulk media. The protonated C8 is able to permeate the cell membrane [16], [18], [50] and release protons into the cell, disrupting the pH gradient. In contrast, HCl is a strong inorganic acid lacking the membrane permeability displayed by the largely hydrophobic carboxylic acid. Thus, HCl is able to pass through the cell membrane only via membrane channels (i.e., ion exchange porins) and proton pumps (e.g., ATPase) [51]. Given these differences, it is unclear if the acid resistance strategies thoroughly described in the literature [35] will also apply to carboxylic acids.

These results highlight the challenge of working with carboxylic acids, as opposed to strong inorganic acids. The membrane permeability of these carboxylic acids interferes with the ability to control intracellular pH within the appropriate range of 6.0–7.5 [52]. Traditional fermentation systems enable maintenance of appropriate media pH, but it is the intracellular pH that impacts productivity. Thus, strategies are needed to combat this acidification effect in order to maintain biocatalyst functionality.

### Native acid resistance systems are ineffective in combatting carboxylic acid stress

Bacterial acid resistance systems have been previously characterized [35]. Specifically there are four known acid resistance systems in *E. coli*. Two of these, acid resistance system 1 (AR1 - *hdeAB* operon and *hdeD*) and acid resistance system 2 (AR2 - *gad* system) all have significantly increased expression in our C8 challenge dataset. Since AR2 is the better characterized [53], [54] of the two, we chose this system as the basis for our attempts to enable increased carboxylic acid tolerance.

AR2 functions by using glutamate as a sink for excess protons, resulting in production of γ-amino butyric acid (GABA) [35], [54]. The fact that the genes encoding this system had increased expression during C8 challenge suggests that *E. coli* is trying to use this system in our condition. This drive to use the glutamate-dependent acid resistance system could explain the observed perturbation of the PutA regulator. AR2 relies on extracellular glutamate for full functionality and thus may have limited effectiveness in our minimal media condition. For this reason, we tested the effectiveness of glutamate supplementation in mitigating both the inhibition of growth and intracellular acidification mediated by octanoic acid.

**Figure 3:**
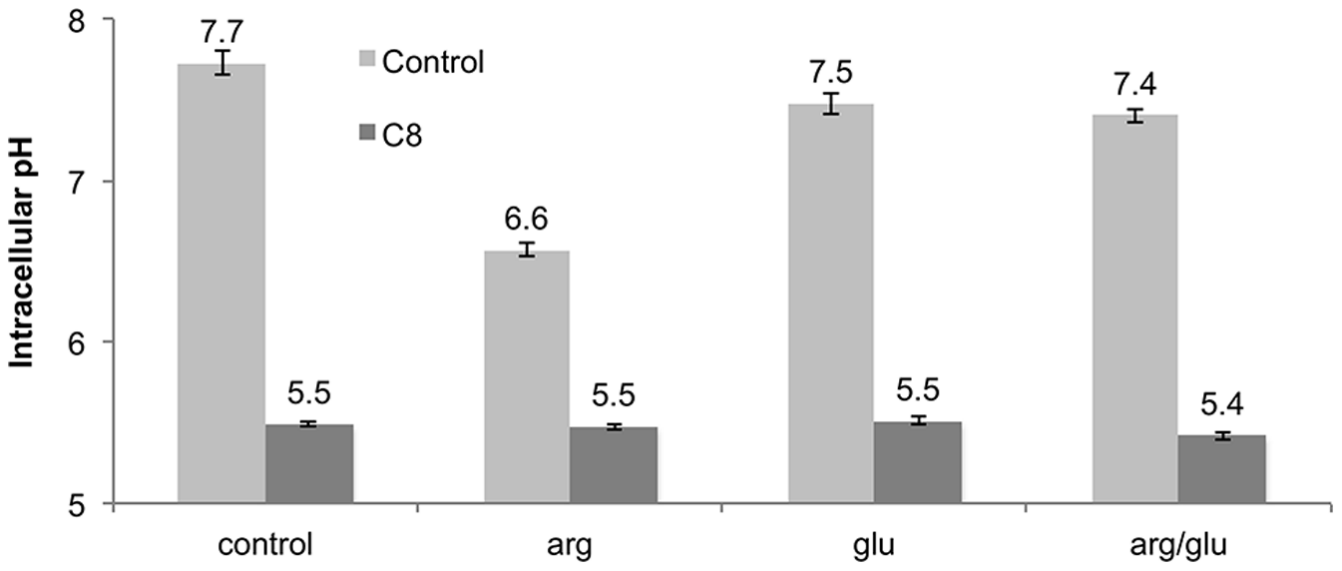
Supplementation with arginine or glutamate does not mitigate intracellular acidification. Measurements of the intracellular pH of E. coli MG1655 pJTD1 during C8 challenge while grown in the presence of supplemental arginine and glutamate. The cells were incubated for 3°C in MOPS media to allow utilization of the amino acid-dependent acid resistance systems. All concentrations are 10 mM.

Supplementation with 10, 20 or 30 mM glutamate was not effective in increasing tolerance to 10, 20 or 30 mM C8 at neutral pH respectively (*unpublished data*). Glutamate supplementation was also not effective at mitigating the acidification effect (Figure 3). Note that the acidification effect presented in Figure 3 is more severe than that shown in Figure 2; this is due to the fact that cells queried in Figure 2 were characterized immediately after C8 addition while cells queried in Figure 3 were incubated in the presence of C8 for 3 hours, providing the opportunity to use the AR2 system. Note that a pH of 5.5 is the limit of detection of the GFP method and therefore the intracellular pH may actually be less than 5.5. We also attempted to utilize acid resistance system 3 (AR3), which relies on arginine as a proton sink. However, supplemental arginine was also ineffective in combating growth inhibition or acidification (Figure 2).

### The glutamate-dependent acid resistance system is impaired during carboxylic acid challenge

The lack of protection conferred by the arginine- and glutamate-dependent acid resistance systems is surprising, especially considering the fact that the expression of the genes encoding the glutamate-dependent system is increased in our dataset. According to the current understanding of the AR2 system, one glutamate molecule can be used as a sink for one intracellular proton. The arginine-dependent system functions similarly. However, neither of these amino acids was able to provide protection in terms of growth or intracellular acidification (Figure 3). To gain insight into this surprising result, we further characterized the activity of the AR2 system.

**Figure 4:**
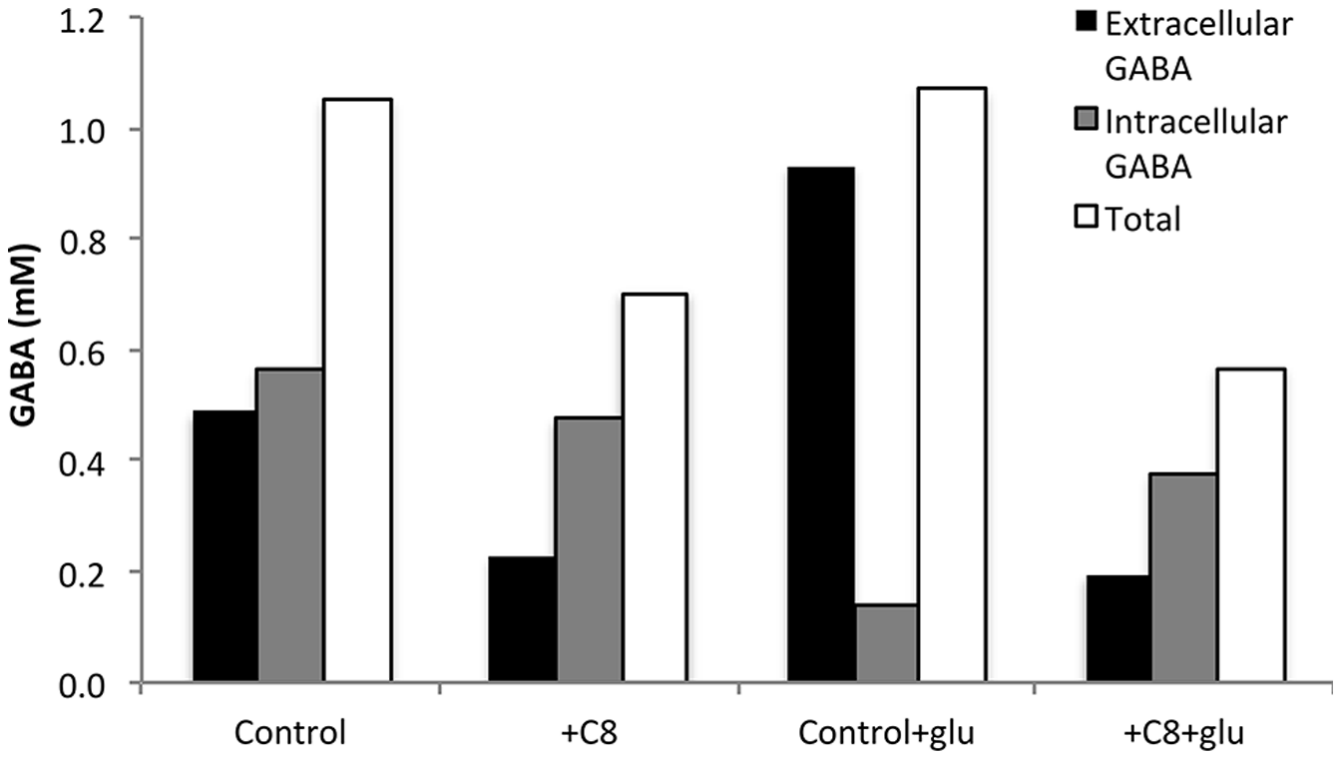
Supplementing glutamate increases GABA export; however, addition of C8 reduces GABA accumulation and export. GABA measurements of MG1655 during log phase growth in MOPS with 2% dextrose at 37°C, 150 rpm. All concentrations are 10 mM. GABA: γ-amino butyric acid.

The product of the proton-dependent glutamate decarboxylation by GadB, GABA, was measured as a reporter of the activity of the AR2 system (Figure 4). GABA is typically released outside of the cell in order to buffer the intracellular pH to physiological levels (∼7.4). This export occurs via the GadC antiport in concert with glutamate import. Note that cells are also capable of transforming GABA into succinate [54]. Therefore, both the internal and external GABA abundance values are meaningful. Internal abundance values reflect GadB activity, internal glutamate availability and the relative abundance of excess protons. External GABA abundance reflects functionality of the GadC antiporter and external glutamate availability.

We observed that in the control condition, the amounts of intracellular and extracellular GABA are relatively equal. While previous studies have measured GABA abundance in *Listeria* [55], *Corynebacterium* [56], *Lactobacillus* [34] and *E. coli* [57], to the best of our knowledge, ours are the first reported measurements for *E. coli* that document steady-state log-phase intracellular and extracellular GABA concentrations.

When supplemental glutamate is added to the control condition, secretion of GABA is increased, though the total amount of GABA remains unchanged relative to the unsupplemented control. This lack of change in the total GABA amount is consistent with the lack of excess protons that need to be removed from the cell interior.

We have already described the fact that addition of 10 mM C8 to the media while maintaining a neutral media pH both impedes growth [5] and severely acidifies the cell interior, with or without provision of 10 mM supplemental glutamate (Figure 3). For the cells challenged with C8 in the absence of glutamate, there was a decrease in intracellular, extracellular and total GABA. For the cells provided with supplemental glutamate during C8 challenge, there was also a decrease in all types of GABA relative to the unsupplemented control. This lack of perturbation to the GABA pools is surprising, given the known excess of intracellular protons in the +C8 condition. Under appropriate functioning, the GadB/GadC system should be producing and exporting GABA as a proton sink. However, this system is apparently not functioning during carboxylic acid challenge.

This lack of function could possibly be attributed to another well-accepted mechanism of carboxylic acid toxicity: membrane damage. Both GadB and GadC have some degree of membrane-associated function [53]. The damage caused to the cell membrane by carboxylic acids can result in impaired function of this system, even when the enzymes and the system precursors, glutamate and excess protons, are abundant. Whereas these two mechanisms were previously thought to be distinct modes of inhibition, to the best of our knowledge, we are the first to link the membrane damage and acidification effect during organic acid challenge. This again highlights the differences in mitigating tolerance to carboxylic acids and strong acids.

### Intracellular acidification is less severe during carboxylic acid production

**Figure 5:**
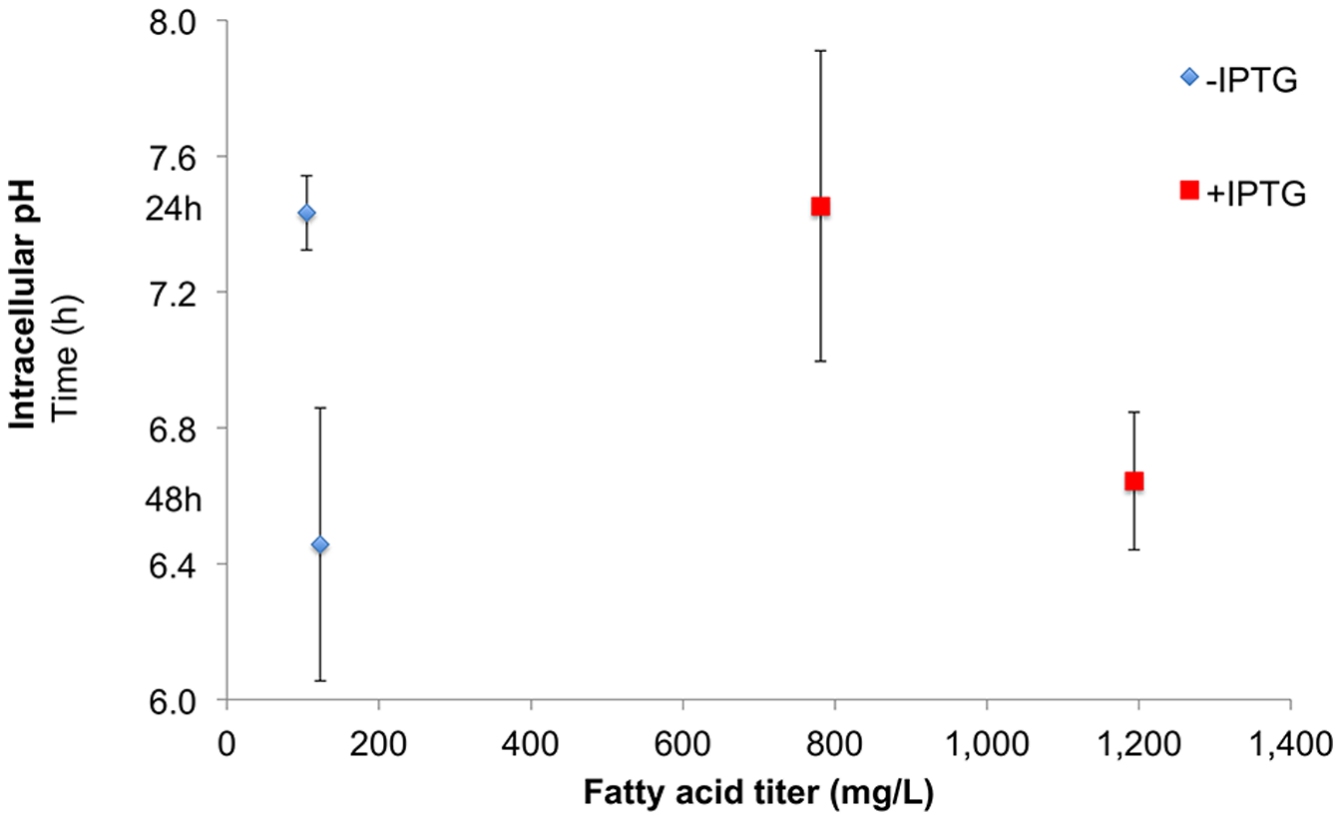
Production of carboxylic acids in strain ML103+pXZ18Z+pZS-GFP does not significantly change the intracellular pH. Shake flasks of E. coli producing predominately C14:0 and C16:0 carboxylic acids in M9 media with 1.5% dextrose at 30°C. IPTG induces the pXZ18Z plasmid carrying a thioesterase and a β-hydroxyacyl-ACP dehydratase. The intracellular pH values are the average of four biological replicates and four technical replicates. The error bars indicate the standard deviation.

Many of the studies of carboxylic acid tolerance are motivated by a desire to produce carboxylic acids at a high titer [9]. Note that some studies are instead motivated by the use of carboxylic acids as food preservatives [58]. The data described above quantifies the intracellular acidification of *E. coli* during exogenous challenge with octanoic acid. However, this response to exogenous challenge does not necessarily correlate to the physiological effects of carboxylic acid production. Therefore, we quantified the intracellular pH during production of carboxylic acids, using a strain that produces a mixture of C14:0 and C16:0 to a titer of roughly 4 mM in minimal media (Figure 5) [21]. Note that this is the same strain for which we observed decreased membrane integrity as product titers increased [5].

While intracellular pH values were observed at or below our reliable detection limit of 5.5 during long-term challenge with octanoic acid (Figure 3), intracellular pH values during carboxylic acid production were above 6.5 (Figure 5). *E. coli* K-12 is generally able to maintain an intracellular pH of 7.6±0.2 when grown in media with a pH between 6.0 and 7.5, as controlled by the addition of ionic buffering agents [59]. Outside of this range, growth is still observed at pH values between 4.5 and 9.0 [59], though growth is inhibited if intracellular pH falls below 7.1 [52]. It has been noted that *E. coli* does not grow if the intracellular pH falls below 6.0 [52]. It is also worth noting that similar intracellular pH values were observed in our control strain, in which transcription of the thioesterase responsible for cleaving the elongating fatty acids was not induced.

This data suggests that unlike carboxylic acid challenge, carboxylic acid production does not result in intracellular acidification. However, the difference in concentration of carboxylic acids may be the main factor responsible for this difference. Our exogenous challenge studies were performed with 10 or 20 mM C8, but the carboxylic acid production strain does not exceed titers of 5 mM. The lack of intracellular acidification during carboxylic acid production could also be due to metabolic effects, as discussed below. To the best of our knowledge, this is the first quantification of intracellular pH during organic acid production.

### Cyclopropane fatty acids as a protectant to low pH

As described above, membrane damage has been previously cited and quantified as a mechanism of biocatalyst inhibition by carboxylic acids [5], [6]. Genes and regulators related to membrane damage were perturbed in our transcriptome data. We have also proposed that membrane damage is the reason for poor functioning of the glutamate-dependent acid resistance system. Previous engineering efforts for increasing carboxylic acid tolerance in *E. coli* have focused on increasing the relative content of saturated lipids in the cell membrane [60]. These efforts were successful in increasing tolerance to carboxylic acids, but not increasing carboxylic acid production.

Given that increased membrane permeability (leakage) is a likely mechanism of carboxylic acid toxicity, it is appealing to implement genetic modifications that increase membrane integrity. Cyclopropane fatty acids are known to have increased bulk in the cell membrane relative to saturated fatty acids [61]. Additionally, cyclopropane fatty acids have been well-documented in the role in conferring resistance to acidic environments [62]. Considering the fact that *E. coli* inhibition by carboxylic acids is associated with both membrane damage and induction of the acid response, we proposed that increasing the abundance of these cyclopropane fatty acids in the cell membrane could serve as a method of increasing carboxylic acid tolerance.

**Figure 6:**
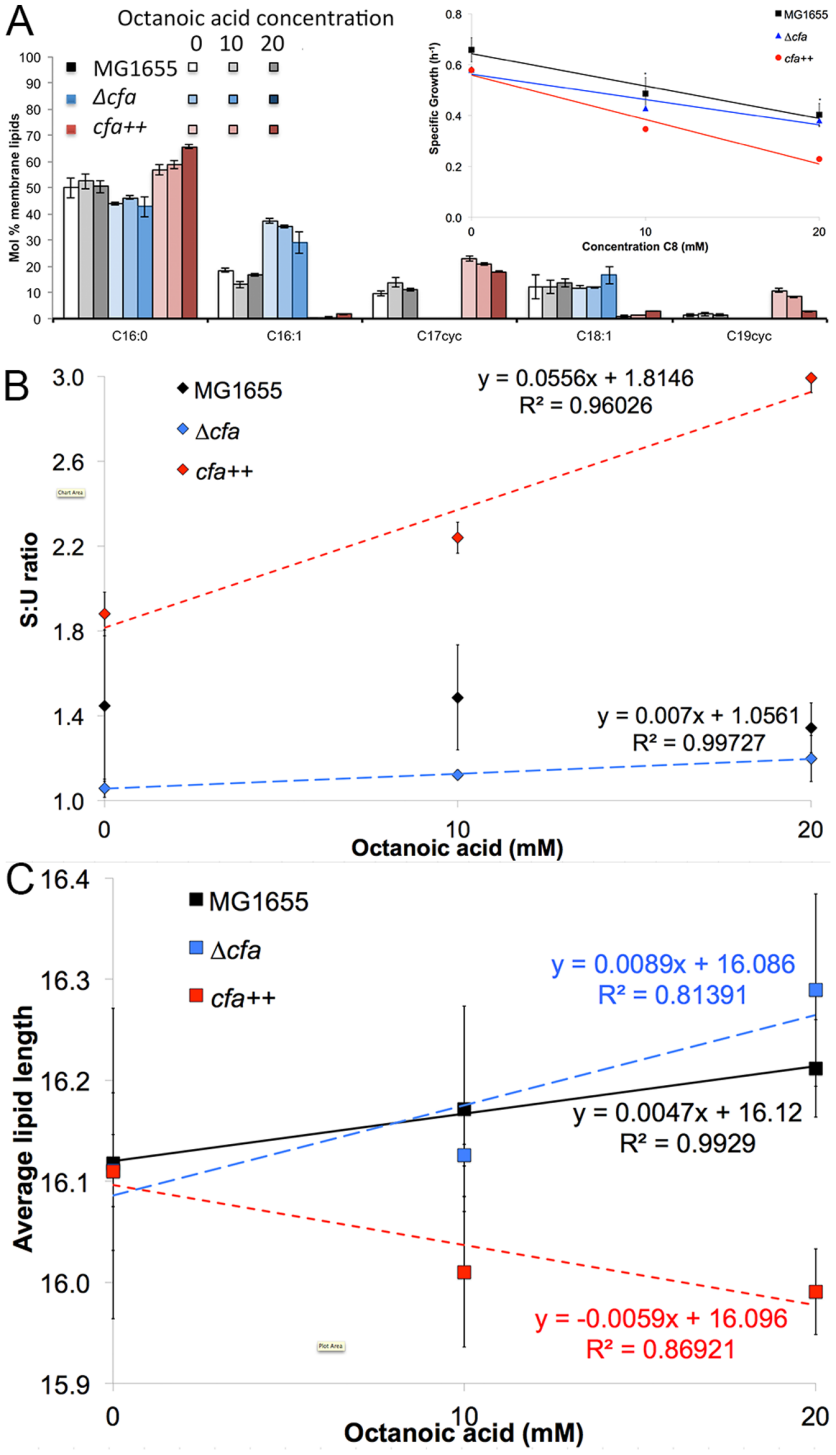
cfa mutants with altered the membrane lipid profile, S:U ratio and the average lipid length. a: Membrane lipid profile of MG1655 and strains with altered cfa expression. Strains were incubated with 0–30 mM C8, pH = 7.0. Inset: specific growth rate in the log phase of E. coli with varying cfa expression. C16:0- palmitic acid, C16:1- palmitoleic acid, C17cyc- cyclopropane C17:0, C18:1- vaccenic acid, C18:0- stearic acid, C19cyc- cyclopropane C19:0. The complete lipid profiles are shown in Figure S2 of the Supporting Information. Membrane properties are calculated from a to obtain: b: saturated:unsaturated lipid ratio and c: average lipid length.

The role of cyclopropane fatty acids in carboxylic acid tolerance was investigated using mutants either completely deficient in cyclopropane fatty acid production (Δ*cfa*) or engineered for increased expression of the Cfa enzyme (*cfa++*). Note that Cfa is responsible for the S-adenosyl-L-methionine (SAM)-dependent methylation of unsaturated membrane lipids [63]. No cyclopropane fatty acids were observed in our Δ*cfa* mutant (Figure 6a). Strains with increased *cfa* expression contained more than 32 mol% cyclopropane fatty acids while less than 11 mol% were observed in the wildtype strain in the control condition (Figure 6a). The complete lipid profiles are shown in Figure S2.

Despite this substantial change in cyclopropane fatty acid content in the cell membrane, there was not an observed increase in C8 tolerance (Figure 6a inset) and no change in leakage or fluidity (*unpublished data*). In fact, strains with the highest cyclopropane fatty acid content actually showed significantly (p<0.05) increased sensitivity to C8 (Figure 6a inset).

Despite the lack of impact on carboxylic acid tolerance, the data obtained in these experiments provides interesting trends in control of membrane composition. We observed previously that when challenged with C8, *E. coli* undergoes a dose-dependent, statistically significant decrease in the molar ratio of saturated (*i.e,*. C16:0) and unsaturated (i.e. C16:1 and C17cyc) membrane lipids (S:U ratio) [5]. Cells deficient in cyclopropane fatty acid production have a saturated:unsaturated ratio in the control condition that is significantly lower than the wildtype strain (Figure 6b). However, as the C8 concentration increases, the S:U ratio in the Δ*cfa* strain approaches the S:U ratio observed in the wildtype strain. Contrastingly, cells with increased cyclopropane fatty acid content (*cfa*++) show a similar S:U ratio as the wildtype strain in the control condition, but when C8 is added at increasing concentration, the observed S:U ratio is increased more than 2-fold relative to the wildtype (Figure 6b). Similar patterns were observed during growth sensitivity measurements. As the C8 concentration increases, the Δ*cfa* strain shows growth rates increasingly similar to the wildtype strain, while the *cfa*++ strain diverges from the wildtype (Figure 6a inset).

Another metric for assessing membrane composition is average lipid length. We previously reported that during growth in the presence of C8, the average lipid length increased in a significant, dose-dependent manner [5]. Cells with increased expression of *cfa* are able to maintain the same average lipid length as the wildtype in the control condition, but in the presence of C8 the lipid length decreases (Figure 6c). This decrease in average lipid length represents a shift from lipids with an 18-carbon chain (C18:0, C18:1, C19cyc) to lipids with a 16-carbon chain (C16:0, C16:1 and C17cyc). Contrastingly, the Δ*cfa* strain followed the wildtype trend by significantly increasing the average lipid length. This was accomplished by decreasing 16:1 and increasing C18:1 (Figure 6a).

Thus, separation of *cfa* expression from the wildtype control circuits impacts not only the saturated:unsaturated fatty acid ratio, but also the average lipid length. This highlights the potential importance of Cfa and cyclopropane fatty acids in maintaining appropriate membrane properties.

### Interaction of Lrp and octanoic acid does not contribute to C8-mediated growth inhibition

It has been previously noted that small carboxylic acids can interact with the global regulator Lrp in a manner than mimics its standard cofactor, leucine [42]. Thus, it is possible that the observed activation of Lrp in our dataset was caused by interaction of Lrp and octanoic acid. Lrp is a global regulator, primarily of amino acid biosynthesis [64] and its activation by a false signal, such as octanoate, could result in inappropriate repression of biosynthesis-associated genes, resulting in growth inhibition.

**Figure 7:**
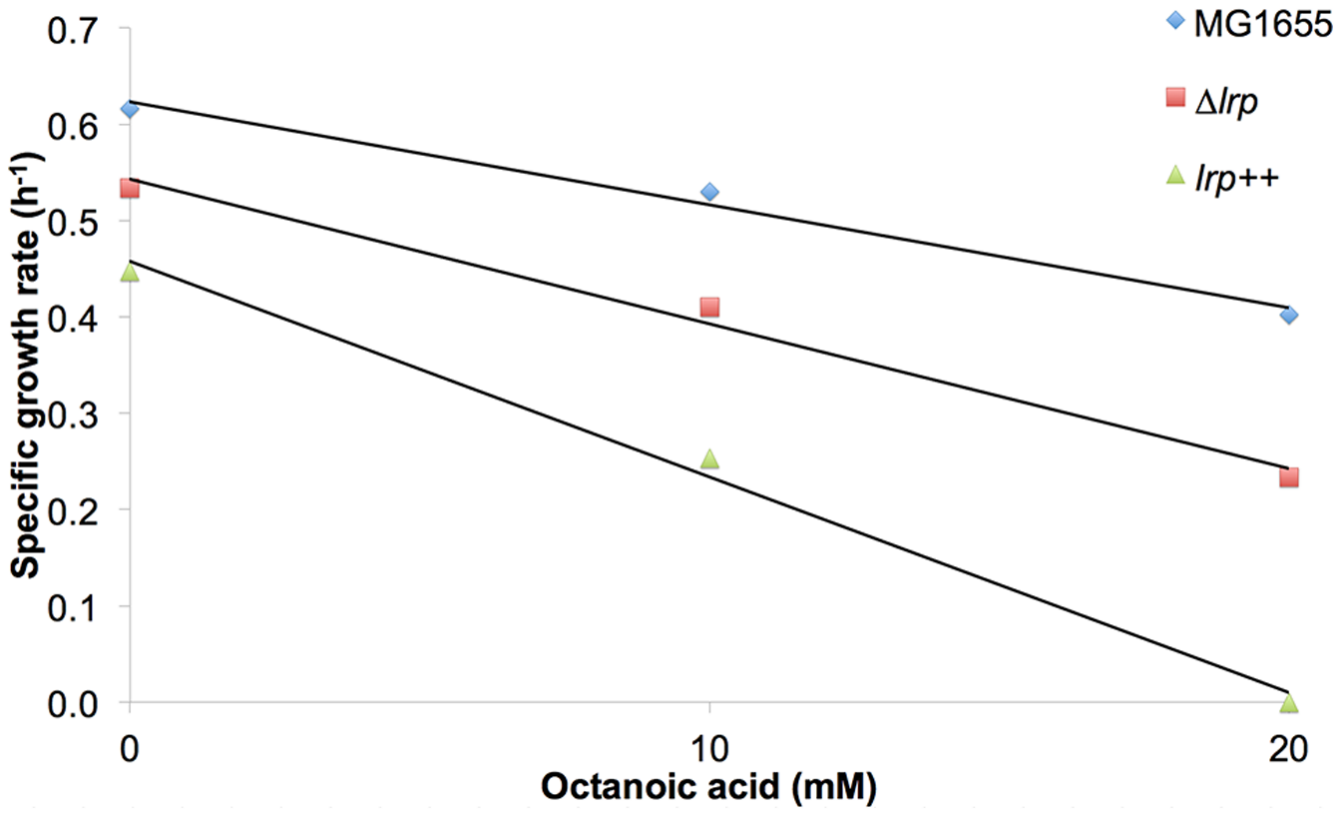
lrp mutations do not improve E. coli growth rate upon addition of octanoic acid. The specific growth rate of strains with altered lrp expression during the log phase in MOPS with 2% dextrose at 37°C, 150 rpm.

We tested the octanoic acid tolerance of both an *lrp* deletion mutant and a strain engineered for increased expression of *lrp*. However, consistent with previous reports [64], the deletion mutant grew poorly in our minimal media growth condition and data regarding carboxylic acid sensitivity was ambiguous (Figure 7). Specifically, the overall growth rate was decreased relative to the wildtype at all C8 concentrations tested, but the relative sensitivity to C8 did not appear to be altered. Similar trends were observed for the strain engineered for increased *lrp* expression (Figure 7).

We proposed that this difficulty in establishing the role of the potential C8-Lrp interaction in C8 tolerance is due to the global regulatory role of Lrp. If C8 is truly serving as a false signal for controlling Lrp activity, this is most likely to impact growth in the form of inappropriate repression of amino acid biosynthesis. Therefore, we tested each of the individual amino acids, as well as a mixture of biosynthetic building blocks in the form of casamino acids, for their ability to confer tolerance to C8. If one or more of these amino acids is limiting during growth in the presence of C8, supplementation with these amino acids should increase C8 tolerance. However, none of the individual amino acid supplements or supplemental casamino acids enabled significantly increased growth in the presence of 10 mM C8 relative to the unsupplemented condition (*unpublished data*). It should also be noted that none of these supplements significantly inhibited growth in the non-C8 condition.

Therefore, even if there is a significant interaction between octanoic acid and Lrp, this interaction does not appear to be contributing to C8-mediated growth inhibition in our condition.

### Octanoic acid causes inversion of the pH gradient leading to anion accumulation in order to maintain the proton motive force

While it does not appear at this point that intracellular acidification is a significant challenge for carboxylic acid production, that perspective may change as we attain higher carboxylic acid titers. Given the qualitative differences observed between challenge with strong inorganic acids and carboxylic acids, we have applied a quantitative modeling approach to identifying additional differences between these two types of acids.

**Figure 8:**
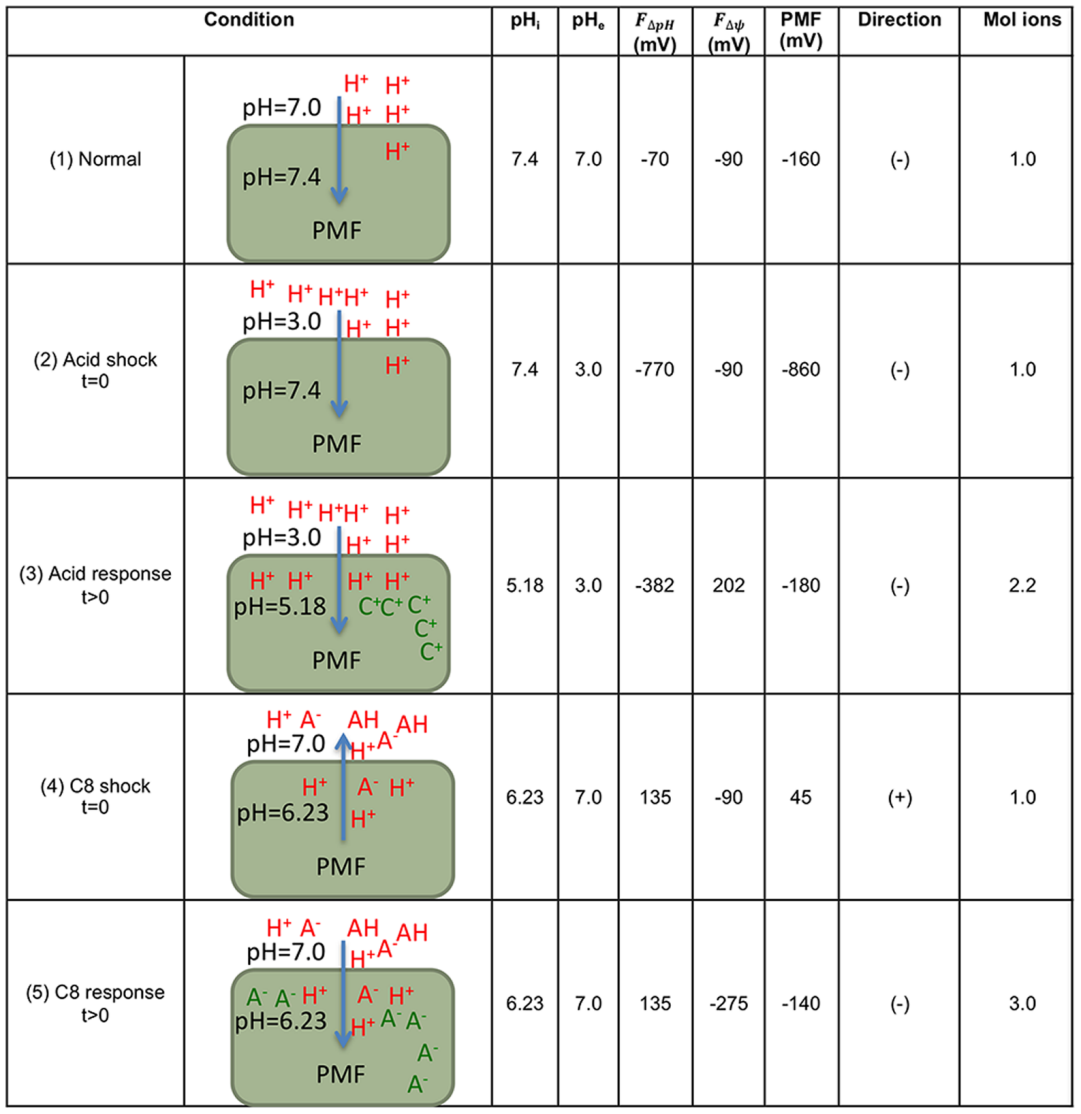
Simulations of the effect of the electrochemical gradients on the proton motive force. The appropriate range for E. coli cells is −140 to −180 mV. pH values are measurements from Figure 2 at 20 mM. Assumption 1: An average of −160 mV is used for the normal PMF. The target PMF is within the range of −140 to −180 mV. Assumption 2: Shock conditions are the instantaneous outcome of the PMF after an extreme change in the environment. Assumption 3: The cell can initiate a response that gives times to adjust the PMF to the target range. Assumption 4: Rapid addition of a strong mineral acid (e.g., HCl) adds an equal amount of anions and protons, which does not change the membrane potential. Assumption 5: C8 is at a sufficiently high concentration that it rapidly integrates into the cell membrane and releases its proton inside the cell, which is not reversible. A negative (−) PMF indicates protons diffusing into the cell, whereas a positive (+) PMF indicates protons diffusing out of the cell. A negative (−)Δψ indicates an overall negative ion gradient due to anions, whereas a positive (+)Δψ indicates an overall positive ion gradient due to cations. The mol ions is the molar ratio from the normal condition. pH_i_- intracellular pH; pH_e_- extracellular pH; 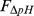
- ΔpH associated electrochemical force; 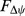
- membrane potential associated electrochemical force; PMF- proton motive force; A^−^ (red) C8 anion; A^−^ (green) another anion; C+ any cation.

The proton motive force (PMF, typically expressed in mV) is a function of both the difference in intracellular and extracellular pH values (ΔpH) and differences in internal and external electrochemical charge (membrane potential, Δψ) as shown above in equation (1). Figure 8 shows our simulations of the pH gradients, membrane potential, and the resultant PMF under different environmental conditions. When cells are shocked with an inorganic acid, such as HCl, the PMF becomes larger than normal and protons initially overwhelm the cell interior. But after a period of adaptation, accumulation of cations can enable reversion of the PMF back to the physiological range, even though the intracellular pH in the adapted state is decreased relative to the original intracellular pH. Such cation accumulation, in the form of potassium, has been previously described for *E. coli* K-12 [17]. This cation accumulation will lead to a change in the transmembrane potential (Δψ) that contributes to stabilization of the PMF. This stabilization of the PMF creates hyperpolarization of the membrane, as evidenced by very large ΔpH and Δψ values. This hyperpolarization can be mediated by the release of chloride anions through voltage-gated channels as discussed in the literature [35], [57], [65], [66]. This type of change in Δψ from negative to positive during challenge with inorganic acids has been previously described [65].

Given the ability of our model to accurately describe the *E. coli* response to inorganic acids, here we have applied this model to carboxylic acid challenge. When cells are shocked with carboxylic acids while maintaining a neutral extracellular pH, the ΔpH value is actually inverted. This inversion of ΔpH results in a transient inversion in PMF. In order to return to the viable PMF range and appropriate directionality, cells must accumulate intracellular anions, as opposed to the cations accumulated after challenge with inorganic acids. Our model predicts that this anion accumulation will increase the intracellular anion pool by 3-fold. This anion accumulation in response to carboxylic acid challenge, as opposed to the cation accumulation seen with inorganic acids, is consistent with literature reports. For example, when *E. coli* O157:H7 was treated with sufficient external acetate to drop the intracellular pH to below 6.5 while maintaining a constant media pH of 5.9, the amount of intracellular acetate increased 3-fold while intracellular potassium levels remained relatively constant [17].

Thus, physiological changes that enable maintenance of the appropriate PMF differ between carboxylic acids and inorganic acids. Specifically, our model predicts that challenge with carboxylic acids will result in a transient inversion of the proton motive force. This prediction is a key component of our analysis, and while it is supported by literature data [17], the existence of this transient inversion is not widely known. Note that *E. coli* K-12 normally maintains a PMF in the range of −140 to −180 mV [35].

## Discussion

While transcriptome analysis is informative about the cellular response network (Figure 1 and Table 2) during challenge with an inhibitor such as octanoic acid, here we have supported these findings with other data that quantitatively describe the physiological state of the cell. We have confirmed that octanoic acid acidifies the cytosol of the *E. coli* cell (Figure 2). Our analysis predicts that this acidification inverts the proton gradient and substantially changes the PMF (Figure 8). Such a change alters the physiological state of the cell in a way that is fundamentally different from inorganic acids such as HCl. Whereas adaptation to HCl includes accumulation of intracellular cations, organic acid stress adaptation includes accumulation of anions in order to revert the PMF to the appropriate range and directionality. Our simulations could be confirmed in future experiments by measuring the intracellular pH, extracellular pH, the transmembrane potential, and intracellular ion concentrations.

We have previously described the negative effects of octanoic acid on the cellular membrane of *E. coli* [5]. Here we have further explored the effect of octanoic acid on membrane proteins such as those associated with acid resistance (Figure 4). While supplementing *E. coli* with glutamate aids in export of the decarboxylation product GABA, it does not support the glutamate-dependent acid resistance system under octanoic acid stress (Figure 3 and Figure 4). We proposed that this system is defective during octanoic acid challenge due to membrane damage.

It is known that cyclopropane fatty acids aid in tolerance of acid stress, presumably by slowing the transport of protons into the cell [62], [63]. Here we have noted that Cfa is involved with complex control of membrane properties, including the saturated:unsaturated lipid ratio and the average lipid length. The effect of Cfa on membrane properties is reflected by the specific growth rate. Our efforts to engineer the membrane in order to confer carboxylic acid tolerance suggests that precise control of the membrane is required for sufficient growth, similar to the conclusions of other researchers [60].

It was reported previously that both exogenous addition and intracellular production of carboxylic acids causes disruption of the membrane [5]. Here we report the differences in how carboxylic acids affect the intracellular pH. While exogenous addition of carboxylic acids causes a significant drop in the intracellular pH, intracellular production of carboxylic acids does not. However, the production strains achieve a much lower titer than the concentrations used in our exogenous challenge studies. It may be that the acidification effect may not become significant in carboxylic acid production strains until higher product titers are achieved. Another potentially-important aspect of carboxylic acid production relates to the distribution of these acids between the protonated and anionic forms within the cell interior. The protonated carboxylic acids can more easily diffuse through the cell membrane than the anionic form [67].

A buildup of intracellular anionic carboxylic acids will shift the equilibrium to the protonated form. The neutral lipophilic fatty acids will then diffuse through the membrane, where it can disassociate, assuming that the media pH is greater than the pK_a_ of the fatty acid.

## Conclusions

These results highlight the ability of transcriptome analysis to guide quantitative experiments in establishing the mechanisms of toxicity of various inhibitory compounds. These mechanisms of toxicity can differ depending on whether the inhibitor is provided exogenously or produced by the cell. Thus, testing of proposed hypotheses in engineered strains is helpful in prioritizing engineering strategies. At this point, it still appears that membrane damage is the most pressing issue limiting carboxylic acid production. Strategies that enable increased membrane integrity may enable increased carboxylic acid production titers, so that intracellular acidification becomes problematic.

## Supporting Information

Figure S1 Transcriptome data of significantly perturbed genes during E. coli challenged with octanoic acid. The criteria for significantly perturbed genes: q<0.05 with a log_2_ ratio <−1.0 or >1.0. Positive values are an increase in abundance and negative values are a decrease in abundance of transcripts. MG1655 was challenged with 10 mM octanoic acid in MOPS with 2% dextrose at 37°C, 150 rpm.
(TIF)

Figure S2 The complete lipid profiles of E. coli strains with engineered cyclopropane fatty acid content. The complete membrane lipid profile of MG1655 and strains with altered cfa expression. Strains were incubated with 0–30 mM C8, pH = 7.0. C12:0- lauric acid, C14:0- myristic acid, C16:0- palmitic acid, C16:1- palmitoleic acid, C17cyc- cyclopropane C17:0, C18:1- vaccenic acid, C18:0- stearic acid, C19cyc- cyclopropane C19:0.
(TIF)

Table S1 Over-represented Gene Ontology terms.Over-represented GO terms
(DOCX)
