# Supplementary figures and images for: Transcriptomic Analysis of Carboxylic Acid Challenge in Escherichia coli: Beyond Membrane Damage
Source: PLoS One. 2014 Feb 28;9(2):e89580. doi: 10.1371/journal.pone.0089580 (PMC3938484; doi:10.1371/journal.pone.0089580)

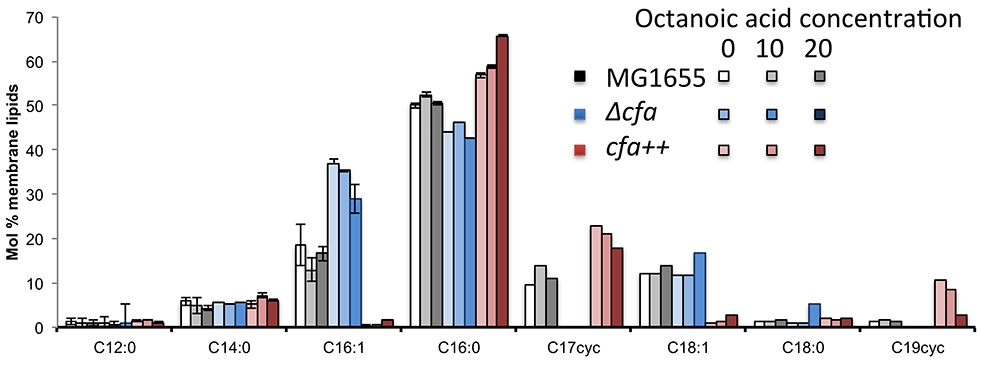

Supplement: Figure S1 — Transcriptome data of significantly perturbed genes during E. coli challenged with octanoic acid. The criteria for significantly perturbed genes: q<0.05 with a log2 ratio <−1.0 or >1.0. Positive values are an increase in abundance and negative values are a decrease in abundance of transcripts. MG1655 was challenged with 10 mM octanoic acid in MOPS with 2% dextrose at 37°C, 150 rpm. (TIF) [file pone.0089580.s001.tif]

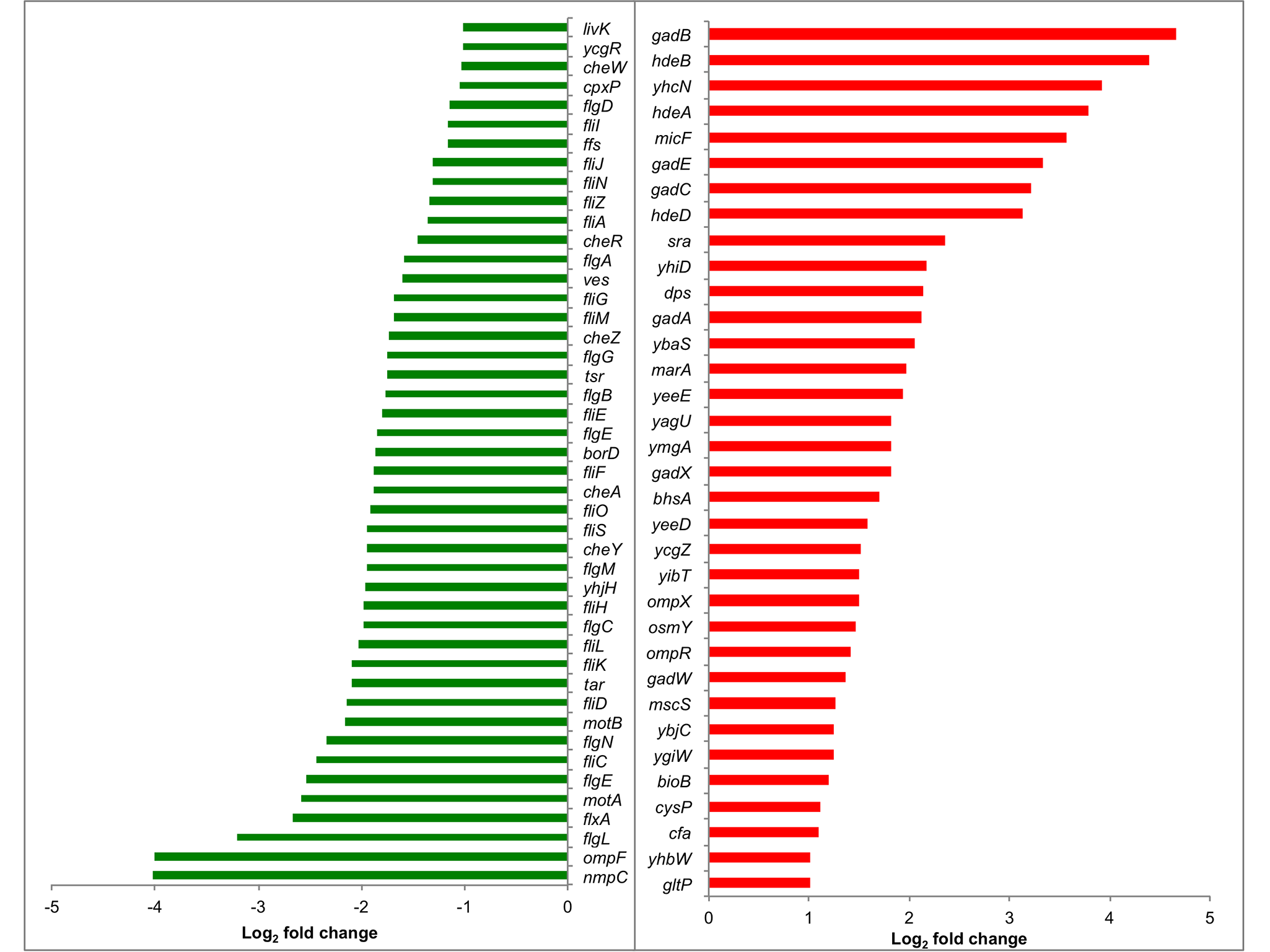

Supplement: Figure S2 — The complete lipid profiles of E. coli strains with engineered cyclopropane fatty acid content. The complete membrane lipid profile of MG1655 and strains with altered cfa expression. Strains were incubated with 0–30 mM C8, pH = 7.0. C12:0- lauric acid, C14:0- myristic acid, C16:0- palmitic acid, C16:1- palmitoleic acid, C17cyc- cyclopropane C17:0, C18:1- vaccenic acid, C18:0- stearic acid, C19cyc- cyclopropane C19:0. (TIF) [file pone.0089580.s002.tif]
