# Supplementary material for: Transcriptomic Analysis of Carboxylic Acid Challenge in Escherichia coli: Beyond Membrane Damage
Source: PLoS One. 2014 Feb 28;9(2):e89580. doi: 10.1371/journal.pone.0089580 (PMC3938484; doi:10.1371/journal.pone.0089580)
Supplement: Table S1 — Over-represented Gene Ontology terms.Over-represented GO terms (DOCX) [file pone.0089580.s003.docx]

# Transcriptomic analysis of carboxylic acid challenge in Escherichia coli: beyond membrane damage

Liam A. Royce^1^, Erin Boggess^2,3^, Yao Fu^2,3^, Ping Liu^4^, Jacqueline V. Shanks^1^, Julie Dickerson^2,3^, Laura R. Jarboe^1,4^

^1^Department of Chemical and Biological Engineering, Iowa State University, Ames, Iowa – 50011, USA

^2^Department of Electrical and Computer Engineering, Iowa State University, Ames, Iowa – 50011, USA

^3^Department of Electrical and Computer Engineering, Iowa State University, Ames, Iowa – 50011, USA

^4^Interdepartmental Microbiology Program, Iowa State University, Ames, Iowa – 50011, USA

To whom correspondence should be addressed: Laura Jarboe, Department of Chemical and Biological Engineering, Iowa State University, 3051 Sweeney Hall, Ames, IA 50011. Telephone: (515)(Polen, 2003 #58) 294 2319, E-mail: [ljarboe@iastate.edu](mailto:ljarboe@iastate.edu)

### Table S1: Over-represented Gene Ontology terms

Over-represented GO terms

| **Type** | **GO term** | **Direction** |
| --- | --- | --- |
| Molecular Function | glutamate decarboxylase activity | up |
|  | sequence-specific DNA binding | up |
|  | structural molecule activity | down |
|  | transmembrane receptor activity | down |
|  | motor activity | down |
| Biological Process | intracellular pH elevation | up |
|  | single-species biofilm formation on inanimate substrate | up |
|  | cellular response to acidity | up |
|  | cellular response to hydrogen peroxide | up |
|  | flagellum assembly | down |
|  | flagellar cell motility | down |
|  | chemotaxis | down |
|  | protein transport | down |
| Cellular Component | periplasmic space | up |
|  | extracellular region | down |
|  | bacterial-type flagellum hook | down |
|  | bacterial-type flagellum basal body, distal rod | down |
